# Trends in antipsychotic polypharmacy and potential overtreatment with antipsychotics- We are not there yet!

**DOI:** 10.1192/j.eurpsy.2025.341

**Published:** 2025-08-26

**Authors:** S. Crutzen, S. Gangadin, K. H. Hua, S. Castelein

**Affiliations:** 1 Lentis Research, Lentis; 2 Psychosis Department; 3 Rob Giel Research Center, University Medical Center Groningen, Groningen; 4 Apotheek Spaarne Gasthuis, Spaarne Gasthuis, Haarlem; 5 Faculty of Behavioural and Social Sciences, University of Groningen, Groningen, Netherlands

## Abstract

**Introduction:**

Due to the potentially severe side effects of antipsychotics, overtreatment is an important concern. Previous research focussed on antipsychotic polypharmacy and excessively high doses.

**Objectives:**

In this study, the aim is to map trends in potential overtreatment, antipsychotic polypharmacy, total antipsychotic dose and the subjective side effect burden. Moreover, the association of the total antipsychotic dose and antipsychotic polypharmacy with the subjective side effect burden will be investigated.

**Methods:**

Data from a large (n>5000) naturalistic longitudinal cohort was used (PHAMOUS, 2013-2021). Potential overtreatment was defined as a total antipsychotic dose equivalent to >5mg risperidone, in combination with a high subjective side effect burden. Mixed effect models were used to investigate trends in potential overtreatment, antipsychotic polypharmacy, total antipsychotic dose and the subjective side effect burden. A mixed effect model was used to assess the association of total antipsychotic dose and antipsychotic polypharmacy with total subjective side effect burden.

**Results:**

Overall, 15,717 observations nested in 5,107 patients were used. About one-third of patients were potentially overtreated, with no change over time. The prevalence of a dose above the equivalent of 5 mg risperidone decreased over time, while antipsychotic polypharmacy prevalence increased. The total subjective side effect burden slightly decreased. A higher dose and antipsychotic polypharmacy was associated with a higher subjective side effect burden.

**Conclusions:**

The subjective side effect burden did decrease the last decade. This might be caused by lower doses and more adequate use of polypharmacy. Still, the overtreatment rate is about one-third and the subjective side effect burden is still high. To reduce the subjective side effect burden and overtreatment, addressing inappropriate antipsychotic polypharmacy remains prudent.

**Disclosure of Interest:**

None Declared

